# Puerarin Prevents Cadmium-Induced Neuronal Injury by Alleviating Autophagic Dysfunction in Rat Cerebral Cortical Neurons

**DOI:** 10.3390/ijms24098328

**Published:** 2023-05-05

**Authors:** Li Wang, Tao Wang, Shuangquan Wen, Ruilong Song, Hui Zou, Jianhong Gu, Xuezhong Liu, Jianchun Bian, Zongping Liu, Yan Yuan

**Affiliations:** 1 College of Veterinary Medicine, Yangzhou University, Yangzhou 225009, China; dx120210188@stu.yzu.edu.cn (L.W.); dx120200175@stu.yzu.edu.cn (T.W.);; 2 Jiangsu Co-Innovation Center for Prevention and Control of Important Animal Infectious Diseases and Zoonoses, Yangzhou 225009, China

**Keywords:** puerarin, cadmium, rat cerebral cortical neurons, autophagic dysfunction

## Abstract

Autophagic dysfunction is one of the main mechanisms of cadmium (Cd)-induced neurotoxicity. Puerarin (Pue) is a natural antioxidant extracted from the medicinal and edible homologous plant *Pueraria lobata*. Studies have shown that Pue has neuroprotective effects in a variety of brain injuries, including Cd-induced neuronal injury. However, the role of Pue in the regulation of autophagy to alleviate Cd-induced injury in rat cerebral cortical neurons remains unclear. This study aimed to elucidate the protective mechanism of Pue in alleviating Cd-induced injury in rat cerebral cortical neurons by targeting autophagy. Our results showed that Pue alleviated Cd-induced injury in rat cerebral cortical neurons in vitro and in vivo. Pue activates autophagy and alleviates Cd-induced autophagic blockade in rat cerebral cortical neurons. Further studies have shown that Pue alleviates the Cd-induced inhibition of autophagosome–lysosome fusion, as well as the inhibition of lysosomal degradation. The specific mechanism is related to Pue alleviating the inhibition of Cd on the expression levels of the key proteins Rab7, VPS41, and SNAP29, which regulate autophagosome–lysosome fusion, as well as the lysosome-related proteins LAMP2, CTSB, and CTSD. In summary, these results indicate that Pue alleviates Cd-induced autophagic dysfunction in rat cerebral cortical neurons by alleviating autophagosome–lysosome fusion dysfunction and lysosomal degradation dysfunction, thereby alleviating Cd-induced neuronal injury.

## 1. Introduction

Cadmium (Cd) is a common environmental toxicant in industry and agriculture. The extensive application of Cd in industry and agriculture has led to its continuous accumulation in soil, water, crops, and other environmental media, and its absorption and accumulation in the body through the food chain [1,2]. It is well known that Cd has a high accumulation rate and a low excretion rate in organisms. Therefore, it easily accumulates in many important organs, including the brain, resulting in multiple organ damage [3,4,5]. Regarding the nervous system, the blood–brain barrier restricts the entry of potentially harmful substances to maintain barrier characteristics [6]. However, under continuous Cd exposure, the integrity of the blood–brain barrier is disrupted, severely affecting nervous system function [7]. In addition, Cd-induced neurotoxicity has been shown to be associated with an increased risk of several major neurodegenerative diseases [8,9,10]. Numerous studies have reported the neurotoxic mechanisms of Cd. Previous studies have shown that Cd exposure induces oxidative stress, apoptosis, and mitochondrial dysfunction in neurons [11,12]. Furthermore, another study showed that in PC12 cells and primary mouse cerebral cortical neurons, Cd exposure impaired autophagic flux, leading to neuronal apoptosis [13]. However, the specific mechanism underlying Cd-induced neuronal autophagic dysfunction has not been fully elucidated.

Autophagy is a dynamic process that depends on lysosomes to degrade and recycle dysfunctional intracellular cytoplasmic components (organelles and protein aggregates). In this process, the autophagosome wraps the substance to be degraded and fuses with lysosomes, causing the autophagosomes to mature into degradable autophagolysosomes [14,15]. The small GTP-binding protein Rab7, SNARE (soluble N-ethylmaleimide-sensitive factor attachment protein receptor (SNARE) protein SNAP29, and HOPS (homotypic fusion and protein sorting) protein VPS41 are involved in this fusion process [15,16]. Rab7, a small GTPase belonging to the Rab family, plays a key role in autophagosome–lysosome fusion events [17,18]. Studies have shown that knockdown of Rab7 leads to the blockage of autophagosome fusion with late endosomes and lysosomes, whereas upregulation of Rab7 improves autophagic flux [19,20]. A previous study showed that Cd inhibits autophagosome–lysosome fusion by preventing Rab7 from recruiting autophagosomes in primary rat proximal tubular cells [21]. In addition, Pue alleviated Cd-induced inhibition of autophagosome–lysosome fusion by restoring Rab7 expression, thereby alleviating Cd-induced autophagic blockade in AML12 cells [22]. The assembly of the SNARE complex is the core mechanism of autophagosome–lysosome fusion [16]. A study showed that in Lund human mesencephalic cells, SNARE protein SNAP29 knockout resulted in LC3II accumulation and the inhibition of autophagic flux. Conversely, SNAP29 overexpression restored autophagic flux and partially rescued PD-related protein alpha-synuclein (α-Syn)-induced autophagic dysfunction and reduced neuronal cell death [23]. Furthermore, the HOPS complex-specific subunit VPS41 is a decisive factor in regulating membrane fusion, which can be independent of HOPS, and has been identified as a potential therapeutic target for PD [24,25]. Studies have shown that defects in VPS41 impair the delivery of HOPS-dependent endocytic cargo to lysosomes, leading to autophagy dysfunction [26,27]. However, the regulatory roles of Pue in SNAP29 and VPS41 remain unclear. In addition, autophagy is considered a protective mechanism against exogenous stimuli [28,29,30,31]. Unlike mitotic cells, which reduce protein pathology by diluting aggregates through cell division, the survival of non-dividing differentiated neurons is highly dependent on autophagy to remove intracellular waste and toxic substances [32]. Studies have shown that autophagic dysfunction plays an important role in cytotoxicity induced by a variety of heavy metals, including lead (Pb), manganese (Mn), molybdenum (Mo), and Cd [33,34,35,36]. Defective autophagosome formation or impaired autophagosome maturation may lead to autophagic dysfunction, causing protein aggregation and ultimately neuronal death [37]. In addition, in a Cd neurotoxicity study, Cd exposure was found to induce autophagy dysfunction in mouse neuroblastoma (Neuro-2a) cells, which in turn led to cell death [38,39]. These studies suggest that correcting autophagic dysfunction may be a potential therapeutic strategy for ameliorating Cd-induced neurotoxicity.

*Pueraria lobata* (Willd.) Ohwi (gegen in Chinese), a medicinal and edible homologous plant in Chinese traditional practice, has been widely used to prepare medicaments and functional foods in recent years [40]. Puerarin (Pue) is the main bioactive component of Pueraria root extract, which can cross the blood–brain barrier and has various biological activities such as anti-inflammatory, antioxidant, anti-apoptotic, and autophagy regulation [41]. Clinically, Pue has been used to prevent and assist in the treatment of various diseases, including cancer, diabetes, diabetic complications, Parkinson’s disease (PD), and Alzheimer’s disease (AD) [42,43]. Studies have shown that Pue can effectively alleviate Cd-induced neuronal oxidative stress and apoptosis [3]. Similarly, Pue had a significant protective effect against Pb-induced nerve cell damage [44,45]. These results indicated that Pue plays a positive role in reducing the toxicity of heavy metals. Further studies have revealed that Pue alleviates Pb- or Cd-induced liver and kidney cell injury by modulating autophagic flux [22,46,47]. However, the role of autophagy in the Pue-mediated alleviation of Cd-induced neurotoxicity has not been elucidated. Therefore, we evaluated the regulation of autophagy by Pue in the process of alleviating Cd-induced neuronal injury in vitro and in vivo, involving the three stages of autophagy, namely activation, autophagosome–lysosome fusion, and autophagosome degradation. Our results further clarified the mechanism by which Pue alleviates the neurotoxicity of Cd through the targeted regulation of autophagy.

## 2. Results

### 2.1. Puerarin Alleviated Cadmium-Induced Neuronal Injury

**Figure 1:**
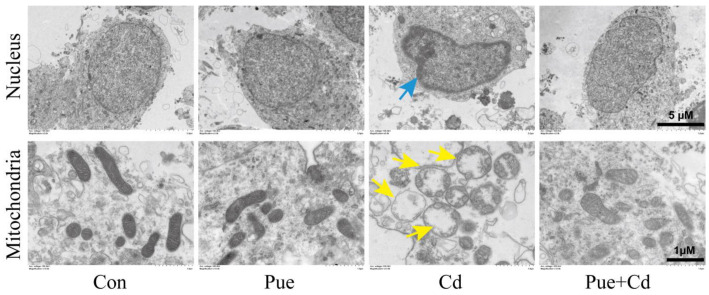
Pue alleviated Cd-induced neuronal injury in vitro. Primary rat cerebral cortical neurons were pretreated with Pue (100 μM) for 1 h and then incubated with or without Cd (10 μM) for 12 h. The nuclei and mitochondria in primary rat cerebral cortical neurons were observed using TEM. The blue arrow indicates the damaged nucleus and the yellow arrow indicates the damaged mitochondria. Scale bar in nuclei: 5 μm; scale bar in mitochondria: 1 μm.

Our previous study showed that Pue can alleviate Cd-induced neuronal damage in vivo and in vitro [3]. On this basis, the potential protective mechanism of Pue was further explored in this study. Firstly, we further confirmed the protective effect of Pue by observing the ultrastructural changes of primary rat cerebral cortical neurons under TEM. As shown in Figure 1, the nuclear membrane was intact, the nuclei were large and round, the matrix was uniform, the mitochondria were relatively complete, and the mitochondrial cristae were clear in the Con and Pue groups. In the Cd-treated group, nuclear membrane deformation, karyopycnosis, chromatin aggregation, mitochondrial crista rupture, and ambiguity presented with obvious vacuolation. Notably, these changes were alleviated in the Pue group. The results confirmed that Pue alleviated Cd-induced injury in primary rat cerebral cortical neurons.

### 2.2. Pue Activated Autophagy and Alleviated Cd-Induced Neuronal Autophagy Blockade In Vitro and In Vivo

**Figure 2:**
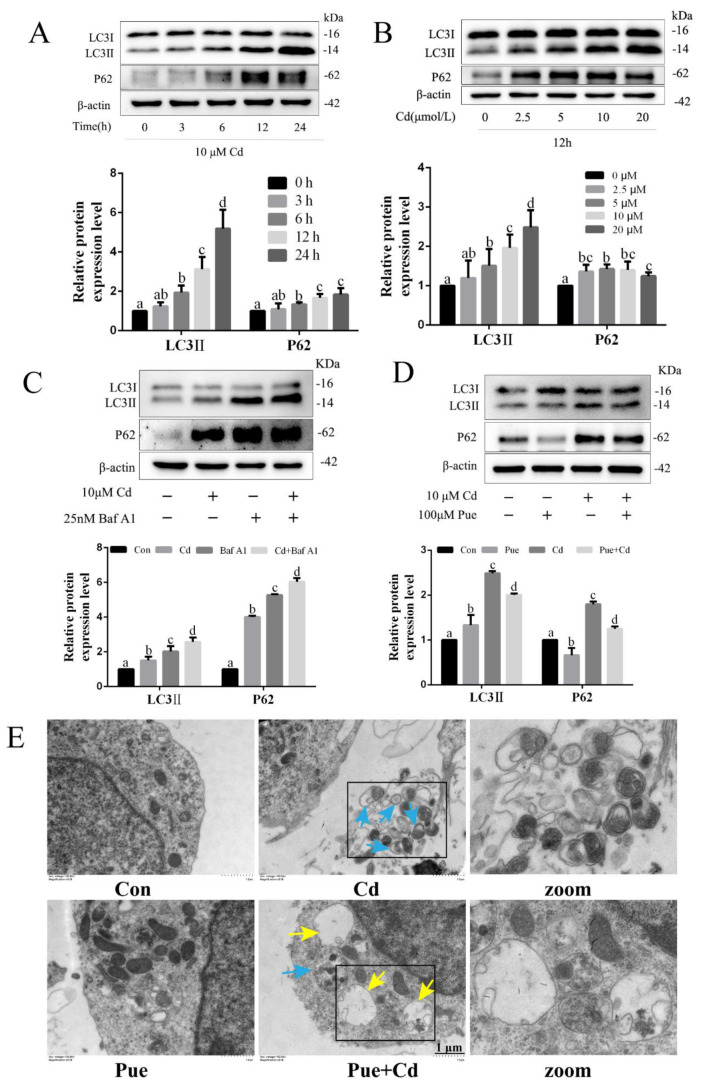
Pue activated autophagy and alleviated Cd-induced autophagic blockade in primary rat cerebral cortical neurons in vitro. The primary rat cerebral cortical neurons were treated with (**A**) 10 μM Cd for different lengths of time (0, 3, 6, 12, and 24 h) or (**B**) with 0–20 μM Cd for 12 h or (**C**) with 10 μM Cd and/or 25 μM Baf A1 for 12 h or (**D**) pretreated with 100 μM Pue for 1 h, followed by 10 μM Cd exposure 12 h. Western blot analysis was then performed to measure LC3II and P62 protein expression. Each value is presented as the mean ± SD (*n* = 3). Completely different letters mean significant differences (*p* < 0.05); the same letters mean no significant differences (*p* > 0.05). (**E**) The representative images of autophagosomes (blue arrows) and autophagolysosome (yellow arrows) in primary cerebral cortical neurons were observed using TEM. The selected areas were magnified. Scale bars: 1 μm.

**Figure 3:**
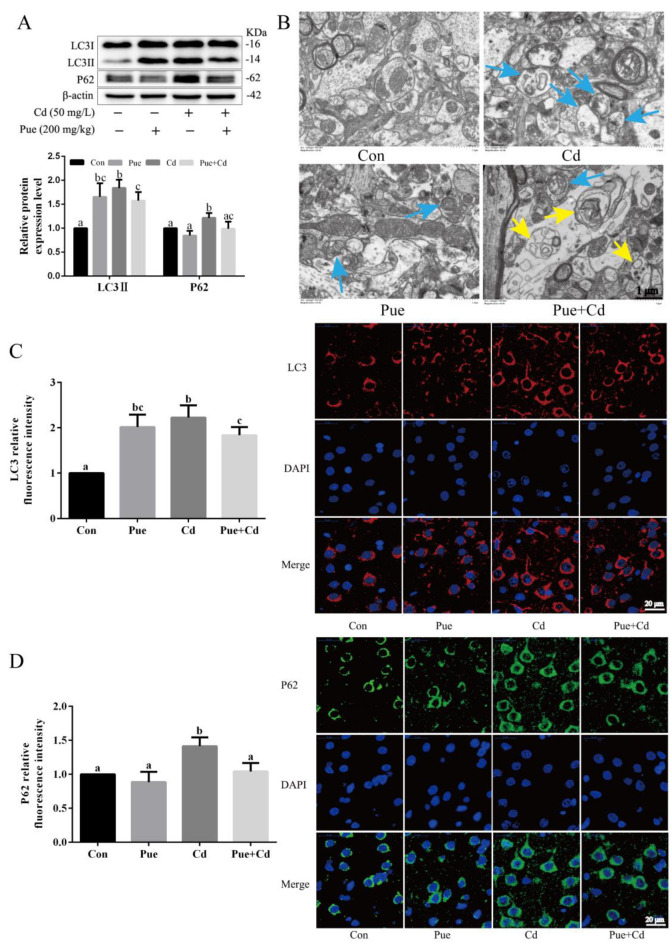
Pue activated autophagy and alleviated Cd-induced autophagic blockade in the rat cerebral cortex in vivo. (**A**) Western blot analysis was performed to measure LC3 and P62 protein expression. Each value is presented as the mean ± SD (*n* = 3). Completely different letters mean significant differences (*p* < 0.05); the same letters mean no significant differences (*p* > 0.05). (**B**) Representative images of autophagosomes (blue arrows) and autophagolysosome (yellow arrows) in the rat cerebral cortex observed by TEM. The selected areas were magnified. Scale bars: 1 μm. (**C**,**D**) Immunofluorescence analysis of LC3 (red), P62 (green), and DAPI (Blue) signals in vivo. Scale bar: 20 μm. The relative fluorescence intensities of LC3 (red) and P62 (green) in vivo were measured by counting 50 cells per condition from three independent experiments (mean ± SD).

Whether Pue alleviates the neuronal damage caused by Cd is related to the regulation of autophagic flux was investigated. The expression levels of autophagy-related proteins were examined using Western blot analysis. As shown in Figure 2A,B, the expression levels of LC3II and P62 showed an overall upward trend with increasing Cd exposure concentration and time. Moreover, the expression levels of LC3II and P62 were simultaneously increased, suggesting that autophagic flux may be blocked. This was subsequently confirmed through the combined use of the autophagy inhibitor bafilomycin A1 (Baf A1), which blocks autophagosome–lysosome fusion and inhibits lysosomal degradation. As shown in Figure 2C, the LC3II and P62 protein levels were significantly increased in the Cd and Baf A1 treatment groups compared with those in the Con group (*p* < 0.05). Furthermore, the LC3II and P62 protein levels in the Cd+ Baf A1 group were further increased compared with those in the Baf A1 alone group (*p* < 0.05), suggesting that autophagic flux was blocked by Cd. Subsequently, compared with the Cd group, the expression of LC3II and P62 was reduced through the combined treatment with Pue and Cd (*p* < 0.05; Figure 2D). Notably, compared to the Con group, the expression level of LC3II was significantly increased in the Pue alone treatment group (*p* < 0.05; Figure 2D). Furthermore, the TEM observation revealed that a large number of autophagosomes with double-membrane structures accumulated in Cd-exposed neurons (Figure 2E). However, in the group treated with Pue and Cd, the number of autophagosomes decreased, whereas the number of autophagolysosomes increased (Figure 2E). Similarly, as shown in Figure 3A,B, in vivo changes in the expression levels of LC3II and P62 proteins and the number of autophagosomes were consistent with the in vitro results. Furthermore, in vivo, the fluorescence changes of LC3 and P62 were observed by immunofluorescence, and the results showed that the fluorescence of LC3 (red) and P62 (green) increased after Cd exposure and decreased after Pue intervention (Figure 3C,D). These results suggest that Pue activates autophagy and alleviates Cd-induced autophagic blockade both in vitro and in vivo.

### 2.3. Pue Attenuated the Inhibition of Neuronal Autophagy–Lysosomal Fusion Induced by Cd In Vitro and In Vivo

**Figure 4:**
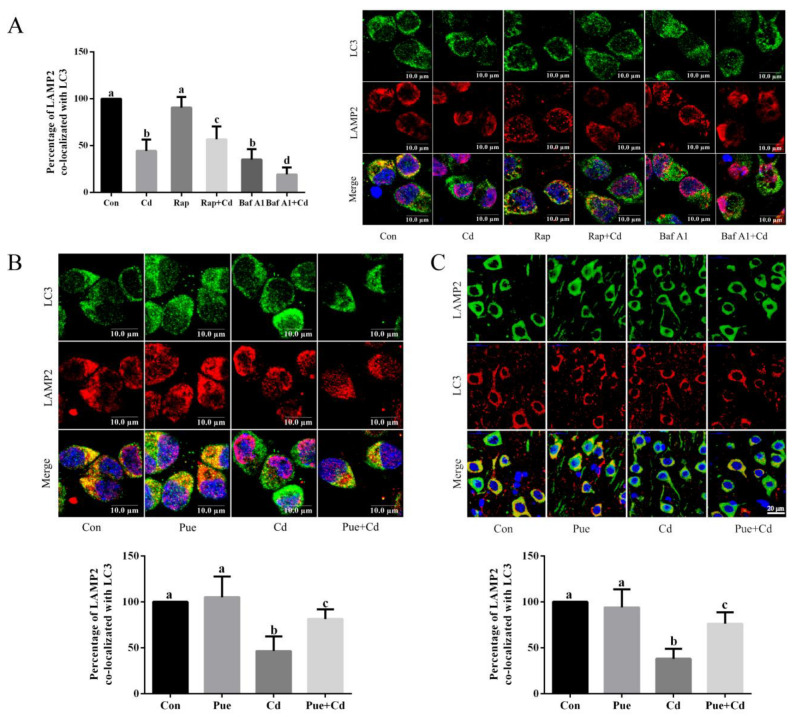
Pue attenuated Cd-induced inhibition of LC3 colocalization with LAMP2 in vitro and in vivo. (**A**) The primary rat cerebral cortical neurons were treated with 10 μM Cd and/or 100 nM Rap (positive control) and 25 nM Baf A1 (negative control) for 12 h. Scale bar: 10 μm. (**B**) The primary rat cerebral cortical neurons grown on coverslips were treated with 10 µM Cd and/or 100 µM Pue for 12 h and then successively stained with LC3 (green), LAMP2 (red), and DAPI (blue). Colocalization of LC3 and LAMP2 was assessed using confocal microscopy. Scale bar: 10 μm. (**C**) The rat cerebral cortices were analyzed for LC3 (red) and LAMP2 (green) colocalization signals using immunofluorescence. Representative confocal images showing colocalization of LC3 with LAMP2. Scale bar: 20 μm. Percent of colocalization of LC3 with LAMP2. The data are represented as the mean ± SD of three independent experiments with 50 cells per condition in each experiment. Completely different letters mean significant differences (*p* < 0.05); the same letters mean no significant differences (*p* > 0.05).

**Figure 5:**
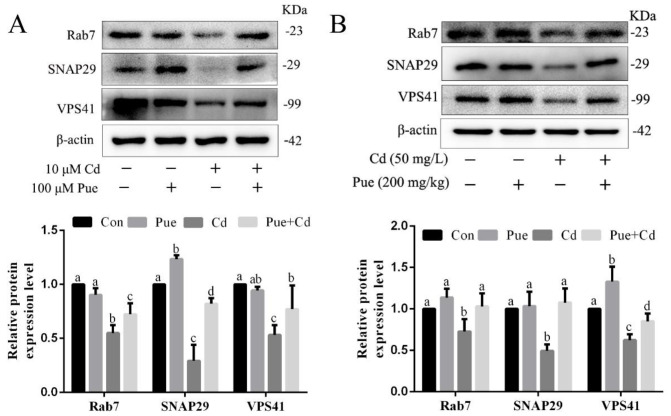
Pue restored the expression of key proteins of autophagosome–lysosome fusion down-regulated by Cd in vitro and in vivo. (**A**) The primary rat cerebral cortical neurons were pretreated with 100 µM Pue for 1 h, followed by 10 μM Cd exposure for 12 h. Then, the expression levels of Rab7, SNAP29, and VPS41 were examined using Western blot analysis. (**B**) The protein samples were prepared from the cerebral cortex homogenate of the rats in each group, and the expression levels of Rab7, SNAP29, and VPS41 were detected using Western blot analysis. Each value is presented as the mean ± SD (*n* = 3). Completely different letters mean significant differences (*p* < 0.05); the same letters mean no significant differences (*p* ˃ 0.05).

To investigate the possible mechanism by which Pue alleviates Cd-induced neuronal autophagic blockade, immunofluorescence labeling of LC3 and LAMP2 was used to assess the autophagosome–lysosome fusion function. In vitro, the autophagy activator Rap was used as a positive control and the autophagy inhibitor Baf A1 was used as a negative control. The results showed that the yellow puncta were more distributed in the Con and Rap groups, less distributed in the Rap + Cd group, and almost absent in the Cd and Baf A1 alone or combined treatment groups (Figure 4A). Furthermore, there were more yellow puncta in the Pue group, and it is noteworthy that the number of yellow puncta in the Pue + Cd group increased compared with that in the Cd group (Figure 4B). Similarly, as shown in Figure 4C, the in vivo changes in the yellow puncta were consistent with the in vitro results. Subsequently, the mechanism by which Pue alleviated Cd-induced inhibition of autophagosome–lysosome fusion was investigated. As shown in Figure 5A,B, the protein levels of Rab7, SNAP29, and VPS41 were significantly downregulated both in vitro and in vivo after Cd exposure compared with those in the Con group (*p* < 0.05). In addition, compared with the Cd group, the expression levels of the above proteins were significantly increased in the combined Pue and Cd treatment group (*p* < 0.05). The above results indicate that Pue alleviated the inhibition of autophagosome–lysosome fusion by Cd, which was related to its regulation of the expression levels of key proteins in autophagosome–lysosome fusion.

### 2.4. Pue Alleviated the Cd Induced Lysosomal Degradation Dysfunction of Rat Cerebral Cortical Neurons

**Figure 6:**
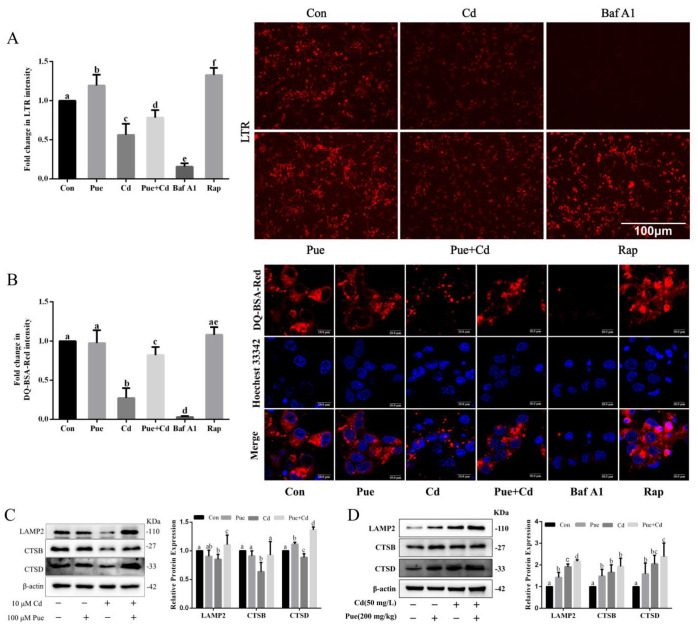
Pue alleviates Cd-induced lysosomal degradation dysfunction in vitro and in vivo. (**A**) The primary rat cerebral cortical neurons were treated with 100 μM Pue and/or 10 μM Cd or 25 nM Baf A1 (negative control) or 100 nM Rap (positive control) for 12 h and then stained with 75 nM LTR and incubated at 37 °C for 30 min to determine the pH of the lysosomes. Scale bar: 100 μm. (**B**) The primary rat cerebral cortical neurons were pre-incubated with 10 μg/mL DQ-BSA Red for 2 h and then treated with 100 μM Pue and/or 10 µM Cd or 25 nM Baf A1 (negative control) or 100 nM Rap (positive control) for 12 h. Hoechest 33,342 staining labeled nucleus (blue). The images were obtained by confocal microscopy. Scale bar: 10 μm. The mean fluorescence intensity of LTR and DQ-BSA Red were quantified in a relative way to the control group, whose value of fluorescence is set at “1”. The data in the ((**A**,**B**) left panel) represent the mean ± SD of three separate experiments and each one was performed in duplicate (*n* = 3). (**C**) The primary rat cerebral cortical neurons were treated with 100 μM Pue and/or 10 μM Cd for 12 h and then the expression levels of LAMP2, CTSB, and CTSD were examined using Western blot analysis. (**D**) The protein samples were prepared from the cerebral cortex homogenate of the rats in each group, and the expression levels of LAMP2, CTSB, and CTSD were detected using Western blot analysis. Each value is presented as the mean ± SD (*n* = 3). Completely different letters mean significant differences (*p* < 0.05); the same letters mean no significant differences (*p* ˃ 0.05).

Further study of the specific mechanism of lysosomal degradation in Pue alleviates Cd-induced autophagy blockade. LTR staining was performed to detect changes in the lysosomal acidic environment. The DQ-BSA fluorescence quenching assay was used to evaluate the lysosomal degradation capacity. Western blotting was used to analyze the expression of lysosome-related proteins in cerebral cortical neurons both in vitro and in vivo. As shown in Figure 6A, Cd exposure increased the pH of lysosomes in primary cortical neurons (LTR red fluorescence decreased) and this change was restored after combined treatment with Pue. These results suggest that Pue effectively prevented lysosomal alkalization of primary cerebral cortical neurons induced by Cd. The results in Figure 6B showed that in the positive control group (Rap group), DQ-BSA was efficiently cleaved, releasing a large number of red fluorescent fragments. Furthermore, compared with the Con group, the red fluorescent fragments of the negative control group (Baf A1 group) and Cd group were reduced, and this change was alleviated in the combined Pue and Cd treatment group. It was suggested that the lysosomal degradation function was damaged after Cd exposure, which could be alleviated by the combination of Pue intervention. As the results in Figure 6C show, Pue alleviated the inhibitory effect of Cd on the expression of lysosome-related proteins LAMP2, CTSB, and CTSD in vitro (*p* < 0.05). However, the in vivo results showed that Pue and Cd exposure, alone or in combination, promoted the expression of lysosome-related proteins. In addition, compared to the Cd group, the Pue + Cd group showed no significant changes in CTSB and CTSD, except for a further increase in the expression level of LAMP2 (Figure 6D). These results suggest that Pue alleviates Cd-induced lysosomal dysfunction in rat cerebral cortical neurons.

## 3. Discussion

The regulation of autophagy as an important protective mechanism of Pue has been reported in studies showing that Pue alleviates Cd-induced liver and kidney cell injury [22,47]. The pharmacological activity of Pue has been shown to have potent protective effects against a variety of brain injuries [41]. However, there is still a lack of research on the mechanism by which Pue regulates autophagy and plays a protective role in Cd-induced neurotoxicity. Therefore, this study further revealed the protective mechanism of Pue-mediated autophagy regulation in vitro and in vivo against Cd-induced cerebral cortical neuronal injury in rats. Our results show that, in addition to activating autophagy, Pue alleviated Cd-induced autophagic dysfunction by restoring autophagosome–lysosome fusion and lysosomal degradation functions, which in turn attenuated Cd-induced neuronal injury in the rat cerebral cortex. This provides a theoretical basis for the application of targeted autophagy strategies in the prevention of Cd-induced brain diseases by Pue and enables Pue preparations to be better applied in clinical practice.

Based on the multi-organ toxicity of Cd, many feasible protection strategies have been explored, and a large number of studies have focused on the role of food nutrients in reducing the toxicity of Cd [48]. Pue is a dietary isoflavone compound, which has attracted much attention because of its potential benefits such as blood–brain barrier protection activity, the reduced toxicity of heavy metals and dietary nutrients, low toxicity, rapid metabolism, and a wide safety range [3,46,49,50]. According to previous reports and our previous experiments, 50–200 μM Pue can alleviate the damage to liver, kidney, and nerve cells induced by heavy metals [3,51,52]. Based on this, 10 μM Cd and 100 μM Pue were used for in vitro experimental research in this study. For the in vivo experiment, we chose drinking water containing 50 mg/L Cd for 90 d combined with 200 mg/kg·bw Pue by intragastric administration [53,54]. Our previously published data have confirmed that Pue can alleviate Cd-induced neuronal damage in vivo and in vitro [3]. This study further confirmed the protective effect of Pue by observing the ultrastructural changes of primary rat cerebral cortex neurons.

Autophagy is an evolutionarily conserved dynamic process that occurs in all eukaryotic cells and has received much attention for its important role in the initiation and progression of a variety of diseases. Studies have found that Cd exposure can induce autophagy in PC12, Sertoli, mouse placenta, and human JEG-3 cells, and the activation of autophagy is beneficial for cell survival [29,30,31]. In addition, a study found that Pue inhibits high glucose-induced podocyte injury by activating autophagy. This protective effect was abolished by the autophagy inhibitor 3-methyladenine [55]. This suggests that the protective effect of Pue is related to autophagy activation. Similarly, in a mouse model of osteoarthritis, Pue exerted a protective effect in chondrocytes by activating Beclin1-dependent autophagy [56]. In addition, the mechanism of the protective effect by activating autophagy has been similarly reported in a model of Pue alleviating Cd-induced hepatocyte injury [22,51]. In this study, analysis of autophagy marker proteins (LC3 and P62) and observation of autophagosomes confirmed that Pue could activate autophagy in rat cerebral cortical neurons. This may be one of the mechanisms by which Pue effectively alleviates Cd-induced injury to cerebral cortical neurons in rats.

Autophagic flux refers to the observed degradation of cytoplasmic material in lysosomes per unit time, and the disruption of autophagic flux leads to autophagic dysfunction [57]. Because autophagy is a dynamic, multi-step process, it is often necessary to coordinate multiple methods for assessment to accurately assess changes in autophagic flux. The combined use of an autophagy inhibitor (Baf A1) to analyze the expression levels of LC3II and P62 is a common method to analyze autophagic flux [58]. In the present study, the combined application of Baf A1 and Cd confirmed that Cd exposure induces autophagy blockade in primary rat cerebral cortical neurons. Blocking autophagic flux is an important cytotoxic effect of Cd. Studies have shown that Cd exposure can induce autophagy blockade in alpha mouse liver 12 (AML12) cells [35], rat proximal tubular epithelial (rPT) cells [59], and PC12 cells [13], resulting in cell damage. Therefore, unblocking autophagic flux may be an effective strategy for alleviating Cd-induced cytotoxic damage. It has been found that Pue can effectively alleviate Cd-induced autophagy blockade in rPT and AML12 cells, thus alleviating cell injury [22,47]. This study further confirmed that in a Cd-induced rat cerebral cortical neuron injury model, Pue could effectively alleviate Cd-induced autophagy blockade both in vitro and in vivo. This indicated that relieving the block of autophagic flux was one of the important mechanisms by which Pue alleviated the Cd-induced injury of cerebral cortical neurons in rats.

Accumulated data indicate that hindered autophagosome–lysosome fusion is one of the main causes of autophagic dysfunction [60,61]. Autophagosomes fuse with lysosomes to form autophagolysosomes, a process regulated by multiple factors such as Rab7, SNARE, and HOPS complexes, ensuring optimal conditions for autophagic flux to proceed [16,62]. Growing evidence suggests that targeting membrane fusion-related molecules, such as Rab7, SNAP29, and VPS41, to modulate autophagy is a viable treatment strategy. In this study, we focused on analyzing the expression levels of the key proteins Rab7, SNAP29, and VPS41, which regulate autophagosome–lysosome fusion. Our results showed that Pue effectively alleviated the inhibitory effects of Cd on the expression of these proteins in rat cerebral cortical neurons. This indicates that Rab7, SNAP29, and VPS41 may be important targets for Pue to alleviate the Cd-mediated inhibition of autophagosome–lysosome fusion in rat cerebral cortical neurons.

Impaired lysosomal degradation is another major cause of autophagic dysfunction. Lysosomal pH, lysosomal membrane proteins, and lysosomal cathepsins are key factors involved in lysosomal degradation [63,64]. LAMP2 is a key membrane protein of lysosomes that plays a key role in maintaining lysosomal pH and lysosomal membrane integrity. CTSB and CTSD are important lysosomal cathepsins involved in lysosomal degradation [64]. Previous studies have shown that the alleviation of lysosomal degradation protects Neuro-2a cells from Cd-induced autophagy dysfunction [38,39]. These results suggest that correcting lysosomal degradation dysfunction during Cd exposure is a promising treatment approach. In addition, another study showed that Cd-induced lysosomal degradation dysfunction in rPT cells could be restored by Pue [47]. Lysosomal degradation functions depend on lysosomal activation. Lysosomal acidification, lysosomal membrane protein expression, and increased lysosomal cathepsin activity are important characteristics of lysosomal activation [65]. Therefore, in the present study, we analyzed the changes in lysosomal activation and lysosomal degradation function in vitro. The results showed that Pue alleviated the Cd-induced inhibition of lysosomal activation and lysosomal degradation in primary rat cerebral cortical neurons. Interestingly, in vivo studies have shown that both Pue and Cd promote the expression of LAMP2, CTSB, and CTSD proteins, suggesting that both Pue and Cd promote lysosomal activation. It has been reported that some lysosomotropic compounds (dimebon, amitriptyline, and verapamil) can evoke compensatory lysosomal activation, but with the ultimate consequence of lysosomal functional impairment. This compensatory lysosomal activation was likely attributed to lysosomal dysfunction, leading to compensatory responses, including the nuclear translocation of the transcriptional factors TFEB, TFE3, and MITF [65]. Similarly, Cd-triggered lysosomal activation in vivo may also be attributable to lysosomal dysfunction, leading to compensatory responses, as the autophagosome accumulation we observed along with the accumulation of the autophagy substrate P62 is strong evidence of lysosomal dysfunction. In addition, unlike Cd-triggered lysosomal compensatory responses, Pue-triggered lysosomal activation has a positive regulatory effect on lysosomes independent of lysosomal dysfunction, and the specific mechanism remains to be further studied. However, the limitation of this study is that the analysis of the whole cortex by Western blotting was unable to assess the difference between neuronal and glial cells, which may be the reason for the difference in the results in vivo and in vitro. In conclusion, these results suggest that lysosomal function plays an active role in defending against the neurotoxic effects of Cd and that Pue plays an active role in the regulation of lysosomal function in Cd-exposed rat cerebral cortical neurons.

## 4. Materials and Methods

### 4.1. Main Reagents

Cadmium acetate (CdAc_2_), Pue (for in vivo studies), and poly L-lysine hydrobromide (L-glutamine) were purchased from Sigma-Aldrich (St. Louis, MO, USA). Sodium Carboxymethylcellulose (CMC-Na) and penicillin–streptomycin were purchased from Solarbio (Beijing, China). Neurobasal^TM^ medium, B-27 supplement, Dulbecco’s Modified Eagle’s Medium/Ham’s F-12 Nutrient Mixture (DMEM/F-12), and fetal bovine serum (FBS) were obtained from Gibco (Grand Island, NY, USA). Rapamycin (Rap), Lyso-Tracker Red (LTR), Hoechst33342 staining, and 4′,6-diamidino-2-phenylindole (DAPI) were purchased from Beyotime (Shanghai, China). DQ-BSA-Green was purchased from Invitrogen (Carlsbad, CA, USA). Pue (for in vitro studies) was purchased from Selleck Chemicals (Houston, TX, USA). Bafilomycin A1 (Baf A1) was purchased from MedChem Express (Monmouth Junction, NJ, USA). All other chemicals and reagents used were of analytical grade.

### 4.2. Cell Isolation and Culture

As previously described [11], the primary rat cerebral cortical neurons were isolated from Sprague Dawley (SD) rats at 18–19 d of gestation by trypsinization and culturing for 6 d for the subsequent experiments.

### 4.3. Animal Administration and Experimental Design

Approval was obtained from the Institutional Animal Care and Use Committee of Yangzhou University; 32 5-week-old male SD rats (140–150 g) were obtained from the Experimental Animal Center of Jiangsu University (Zhenjiang, China). All of the rats were housed in animal rooms with a controlled temperature (23 ± 2 °C), 50–60% relative humidity, and regular light (12/12 h light/dark cycle). After 1 week of adaptation to these conditions, the rats were randomly allocated into four groups: control (Con), Pue, Cd, and Pue + Cd groups. The rats in the Pue and Pue + Cd groups were gavaged with Pue (200 mg/kg·bw) dissolved in 5 g/L CMC-Na solution daily, and the rats in the Con and Cd groups were gavaged with the same amount of CMC-Na solution (5 g/L) daily. From the 15th day, the treatment methods of the Con and Pue groups remained unchanged, and the rats in the Cd and Pue + Cd groups were given drinking water containing Cd (50 mg/L) at the same time as gavage for 90 d. After 90 d, all of the rats were sacrificed under deep anesthesia. Whole brain tissue was collected and fixed in 4% paraformaldehyde to observe the LC3 and P62 fluorescent foci and the colocalization of LC3 and LAMP2 in the rat cerebral cortical neurons. Some tissue was fixed in 2.5% glutaraldehyde and used to observe the ultrastructure, autophagosome, and autophagolysosome changes in the rat cerebral cortical neurons. The remaining tissues were stored at −80 °C to detect changes in related protein levels.

### 4.4. Analysis of Transmission Electron Microscopy (TEM)

The primary rat cerebral cortical neurons were seeded at a density of 7.5 × 10^6^ cells in 10 cm dishes and cultured until day 6. Subsequently, the cells were pretreated with 100 μM Pue for 1 h, followed by 10 μM Cd alone or in combination for 12 h. Then, the cells were collected and fixed with 2.5% glutaraldehyde overnight. The cells were then dehydrated in graded ethanol and embedded in epoxy resin. The sections were sectioned with a microtome and stained with uranyl acetate and lead citrate. Finally, the sections were observed using an HT7800 TEM (Hitachi, Tokyo, Japan).

### 4.5. Western Blot Analysis and Antibodies

The collected primary cerebral cortical neurons and cerebral cortical tissue were lysed in RIPA buffer supplemented with a protease inhibitor cocktail and incubated for 30 min on ice. The lysates were sonicated for 15 s, centrifuged at 12,000 r/min for 10 min at 4 °C, the supernatant was collected, and the total protein was quantified using a BCA assay. Equal amounts of protein (25 μg) were separated using 8–12% sodium dodecyl-sulfate polyacrylamide gel electrophoresis (SDS-PAGE) and then transferred onto a polyvinylidene difluoride (PVDF) membrane. Afterward, they were blocked with 5% non-fat dry milk for 1.5 h at room temperature and incubated overnight at 4 °C with the following primary antibodies: LC3B (1:1000, Cell Signaling Technology, 83506), P62 (1:1000, Sigma, P0067), Rab7 (1:1000, Cell Signaling Technology, 9367), SNAP29 (1:1000, Abcam, ab181151), β-actin (1:1000, Cell Signaling Technology, 4970), VPS41 (1:1000, Santa Cruz Biotechnology, sc-377118), LAMP2 (1:500, Sigma, L0668), Cathepsin B (1:1000, CTSB, Cell Signaling Technology, 31718), and Cathepsin D (1:500, CTSD, Santa Cruz Biotechnology, sc-377124). The following day, the membranes were incubated with anti-mouse or anti-rabbit secondary antibodies for 2 h at room temperature, washed with TBST, and detected using electrochemiluminescence. ImageJ software (V1.53) was used to quantify the protein levels. The density of each band was normalized to that of its respective loading control (β-actin).

### 4.6. Immunofluorescence Analysis of Primary Cerebral Cortical Neurons

The cerebral cortical neurons were plated at a density of 2 × 10^5^ cells on 0.01% poly L-lysine-coated sterile coverslips. After treatment, the cells were fixed with 4% paraformaldehyde for 20 min, permeabilized with 0.3% Triton X-100 at room temperature for 20 min, blocked dropwise with 5% bovine serum albumin (BSA), and incubated at 37 °C for 30 min. The fixed cells were incubated with mouse anti-LC3B (1:300) or rabbit anti-LAMP2 (1:100) overnight at 4 °C. The slides were then washed three times with phosphate-buffered saline (PBS) and incubated at room temperature with Alexa Fluor 488 or a cy3-labeled secondary antibody (Beyotime, Shanghai, China) at a 1:200 dilution based on the source of the primary antibody. Finally, the cells were stained with DAPI, observed, and photographed using a laser confocal microscope (Leica, Wetzlar, Germany). The images for the colocalization analysis (colocalization of LC3 with LAMP2) were calculated using the JaCoP plugin in Image J after the thresholding of individual frames. The colocalization analysis was performed on three independent studies with 50 cells per condition in each experiment.

### 4.7. Immunofluorescence Analysis of the Rat Cerebral Cortex

After the rat cerebral tissue sections were deparaffinized with xylene and dehydrated with gradient ethanol, they were placed in a microwave oven for antigen retrieval. The cells were blocked with 3% BSA at room temperature for 30 min. Then, the slices were placed flat in a wet box and incubated overnight at 4 °C with the primary antibody dropwise. The primary antibodies used were mouse anti-LC3B (1:300), rabbit anti-P62 (1:100), or rabbit anti-LAMP2 (1:100). After the slides were washed trice on a decolorizing shaker, a fluorescent secondary antibody of the corresponding species was added dropwise to cover the tissue and incubated at room temperature in the dark for 50 min. The nuclei were counterstained with DAPI staining solution for 10 min at room temperature. The sections were then mounted with an anti-fluorescence quencher and observed under a laser confocal scanning microscope, and images were collected. The images for the colocalization analysis (colocalization of LC3 with LAMP2) were calculated using the JaCoP plugin in Image J after the thresholding of individual frames. The data are derived from three independent experiments and six random view fields per animal.

### 4.8. Analysis of DQ-BSA Staining

The primary cerebral cortical neurons (2 × 10^5^ cells/well) in 24-well plates were incubated with DQ-BSA Red (10 μg/mL) for 2 h in advance and then incubated with Pue (100 μM) and/or Cd (10 μM), rapamycin (Rap) (100 nM), and bafilomycin A1 (Baf A1) (25 nM) for 12 h. Hoechst 33,342 was stained for 5 min and observed under a confocal microscope.

### 4.9. Analysis of Lyso-Tracker Red Staining

The primary cerebral cortical neurons in 24-well plates were treated with Pue (100 μM), Cd (10 μM), Rap (100 nM), and Baf A1 (25 nM) for 12 h and then incubated with preheated Lyso-Tracker Red (LTR, 75 nmol/L) for 30 min at 37 °C to label the lysosomes. The fluorescence signals of the LTR were observed using an inverted phase-contrast microscope (Leica, Germany).

### 4.10. Statistical Analysis

Data from at least three independent experiments were analyzed. Statistical tests were performed using one-way analysis of variance (ANOVA) with spass 22.0. Multiple comparisons between the groups were performed using the least significant difference (LSD) and Dunnett’s method after determining the homogeneity of variance among the groups. The statistical results were expressed as the mean ± standard deviation (SD). Statistical significance was set at *p* < 0.05.

## 5. Conclusions

In summary, our results suggest that Pue can reduce the neurotoxic effects of Cd by regulating autophagy through multiple targets, mainly by alleviating autophagosome–lysosome fusion dysfunction and lysosomal degradation dysfunction. These findings further enrich the neuroprotective mechanism of Pue and provide theoretical support for the study and application of autophagy-targeting strategies to protect against Cd poisoning.

## Data Availability

The data presented in this study are available upon request from the corresponding author, Y.Y.
